# Case Report: Wide spectrum of *SALL1* variants—a rare cause of pediatric chronic kidney disease

**DOI:** 10.3389/fped.2025.1649707

**Published:** 2025-09-08

**Authors:** Martina Filipič, Špela Stangler Herodež, Mirjam Močnik, Sonja Golob Jančič, Nataša Marčun Varda, Danijela Krgović

**Affiliations:** ^1^ Department of Pediatrics, University Medical Centre Maribor, Maribor, Slovenia; ^2^ Laboratory of Medical Genetics, University Clinical Centre Maribor, Maribor, Slovenia

**Keywords:** chronic kidney disease, child, genetics, rare variants, Townes-Brocks syndrome

## Abstract

**Introduction:**

Genetic causes of chronic kidney disease present a diverse group. Some of them are associated with extrarenal malformations, especially ear anomalies. Genetic diagnosis is essential to confirm the diagnosis, search for additional potential manifestations, and predict the prognosis.

**Case presentation:**

We report a case of a 10-year-old girl with elevated creatinine in whom the presence of auricular appendices led to clinical suspicion of a genetic cause of chronic kidney disease. After investigation, Townes-Brocks syndrome was confirmed with the absence of anorectal and limb abnormalities. Therefore, it can be less apparent and needs to be suspected of.

**Conclusion:**

Rare causes of renal diseases can be suspected already after examination. Malformations of the ears are particularly associated with chronic kidney disease, which should be screened for in such cases. Genetic confirmation allows for the establishment of diagnosis and comprehensive management.

**Summary:**

Townes-Brocks syndrome highlights the importance of chronic kidney disease suspicion in a child when malformations of the ears are present in a child.

## Introduction

Genetic diseases and syndromes often affect the kidneys and lead to chronic kidney disease (CKD), which is present in approximately 10% of the world's adult population and, according to recent data, in 1% of the child population (1, 2). The general opinion that genetic and environmental factors are important in the development of CKD has been reinforced in recent years by the rapid progress in understanding the genetic basis of kidney function and disease (1). Today, more than 600 genes are known whose mutations are involved in the development of monogenic kidney disease (3). In the pediatric population, excluding diabetic nephropathy and hypertension, these genes are responsible for 20%–50% of CKD cases (1, 4). In the European study of congenital anomalies of the kidneys and urinary tract (CAKUT), in cases with extrarenal manifestations, the latter were part of known syndromes or chromosomal abnormalities in more than half of the cases (5).

Abnormalities affecting both the kidneys and the ears are among the most frequently observed associations and were first recognized around 80 years ago. The association is not clear in all cases, but mostly it includes mutations in transcription factors, growth factors, and their receptors affecting the differentiation of both kidneys and ears (6).

Individuals with ear malformations have a higher incidence of clinically significant structural renal anomalies compared to the general population (6). Kidney disorders linked to ear abnormalities encompass a broad spectrum, including glomerulopathies, CAKUT, ciliopathies, and tubulopathies (7). Therefore, whenever ear abnormalities are present, kidney disease should be searched for.

## Case description

A 10-year-old girl came to our department for an additional diagnostic workup of suspected CKD. Before that, she was found to have an elevated creatinine level (88 μmol/L) a few days after an episode of gastroenteritis. However, with additional medical documentation review, we found that her creatinine levels were elevated at a similar level (85 μmol/L) already two years before that episode with no apparent cause. CKD was suspected, and additional diagnostics was performed.

Her family members were unaffected concerning kidney disease. She did have problems with a cough that might be of gastroesophageal origin. Also, she had a history of allergies (cat hair, pollen, grass) and lactose intolerance. In her physical examination, we noticed that she wore glasses; otherwise, no significant findings were seen at the general examination, such as anorectal, limb, or ear anomalies. Her physical growth was unaffected; at 10 years of age, she was 146 centimeters high reaching 90th percentile and weighed 37 kilograms reaching 75th percentile, both percentiles evaluated according to CDC growth charts.

In the laboratory work-up, we confirmed elevated serum creatinine level (94 μmol/L) with elevated cystatin C (1.45 mg/L) and normal urea and electrolytes. In the urine, there was no significant daily proteinuria (less than 0.17 g/day), no hematuria, only mild microalbuminuria (urinary albumin/creatinine was 4.3 g/mol), and a slightly increased amount of low-molecular-weight proteins in the morning urine sample (urinary ɑ-1-microglobulin/creatinine was 2.24 g/mol). Ultrasonographically, both kidneys were hypoplastic with preserved corticomedullary differentiation and without other morphological pathology such as obstructive uropathy or vesicoureterorenal reflux. Her right kidney was measured at 6.8 centimeters and left at 7.2 centimeters, both under 1st percentile for her age.

Glomerular filtration rate was assessed using iohexol, which showed glomerular filtration of 61 ml/min/1.73 m^2^, confirming stage 2 CKD. In accordance with her stage, other complications, such as anemia, high blood pressure, proteinuria, and poor nutrition, were searched for and excluded at the time. Further follow-up is needed to monitor her CKD progress.

**Figure 1:**
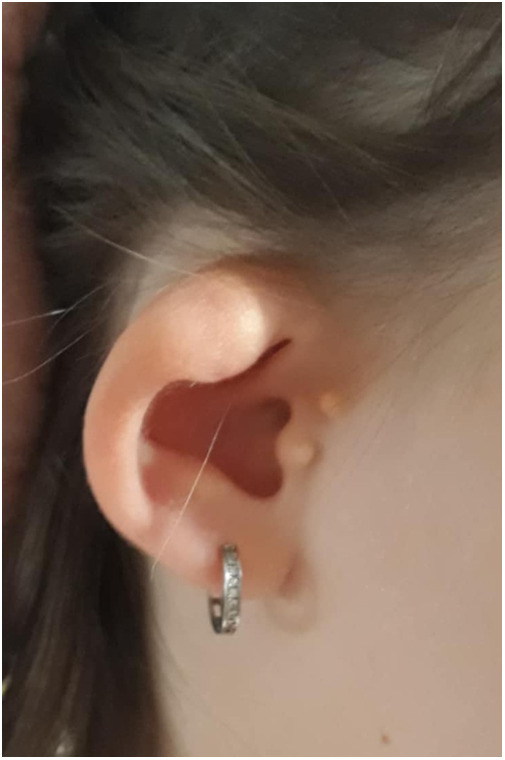
Preoperative view of the patient's hypoplastic ear with appendages.

Meanwhile, we also performed a more thorough medical history, where we found out that the girl's periauricular appendages were removed at the age of 5, and hypoplastic ears (photo documentation from the mother before the operation presented in Figure 1) were operated on. Her audiometry showed mild bilateral sensorineural hearing loss with hearing threshold 20–30 dB. According to her presentation we suspected a genetic origin of her CKD with extra-renal manifestations in the branchio-oto-renal (BOR) spectrum. Her blood sample was sent for genetic testing using targeted panel sequencing (next-generation sequencing (NGS) of genes *EYA1*, *SIX1*, *SIX5*) coupled with multiplex ligation dependent probe amplification (MLPA) using the SALSA MLPA Probemix P153 EYA1 (https://www.mrcholland.com), which yielded no genetic mutations.

Additionally, whole exome sequencing (WES) of the patient and parents was performed, which showed the presence of a *de novo* pathogenic heterozygous insertion NM_002968.3:c.1148_1149insTTTA in the *SALL1* gene that caused the insertion of four nucleotides, resulting in a frameshift change from 383. amino acid. This variant has not been described previously and is considered pathogenic (class 5) according to American College of Medical Genetics and Genomics (ACMG) guidelines (8).

Therefore, we conclude that in our patient, a novel mutation in the *SALL1* gene is a cause of Townes-Brocks syndrome, diagnosed in our patient.

## Discussion

More than 20 disorders encompass both kidney and ear abnormalities (7). Commonly, the ears are affected mainly by sensorineural hearing loss, as in the most commonly associated Alport syndrome; however, in some cases, external ear malformations are present, such as in branchio-oto-renal (BOR) syndrome, CHARGE syndrome, and Townes-Brocks syndrome (7). These are usually recognized soon as several other malformations are present as well. The least obvious is BOR syndrome, usually characterized by branchial fistulas, hearing impairment, renal malformations, and auricular anomalies (9). It has a high penetrance with variable expressivity (9); therefore, it was also the first differential diagnostic option in our case.

CHARGE and Townes-Brocks syndrome were less likely due to the absence of other manifestations. CHARGE syndrome is a genetic condition characterized by a specific and recognizable pattern of features: coloboma, heart defects, choanal atresia, retardation of growth or development, genitourinary malformations, and ear anomalies. Consequently, it was less likely in our case; however, the phenotypic spectrum associated with variants in the chromodomain helicase DNA-binding protein 7 (CHD7) as the major cause of CHARGE syndrome has been broadened. Several predicted pathogenic *CHD7* variants have been identified in individuals with isolated features of CHARGE syndrome (10); therefore, this was also possible in our case. Townes-Brocks syndrome is characterized by the triad of anorectal malformations, dysplastic ears, with or without hearing impairment, and hand or thumb anomalies (11), which seemed less likely, since in our case there was no anorectal or hand and thumb abnormalities.

After exclusion of BOR syndrome, we therefore proceeded with WES testing of patient and parents, which revealed a *de novo* pathogenic mutation in *SALL1* gene, usually consistent with Townes-Brocks syndrome, however with absence of cardinal features, such as anorectal and limb malformations. Mutations in *SALL1* gene with atypical presentation were first described 25 years ago (12), followed by other similar, but rare, reports (13). Both reports present similar clinical manifestations as in our case. The entity was also named Townes-Brocks like syndrome in older publications or Townes-Brocks syndrome variant or *SALL1* variant in recent publications and includes hypoplastic kidneys, kidney function impairment, gastroesophageal reflux, ear abnormalities, mild developmental delay, hearing loss and vision impairment, with the absence of rectal and limb abnormalities, as usually typical for Townes-Brocks syndrome. Our patient showed almost all manifestations of Townes-Brocks syndrome or *SALL1* variant; even her need for glasses, and history of persistent cough that was explained with gastroesophageal reflux, as part of her genetic disease, leading to limiting further diagnostic work-up and burden in persistent cough evaluation. Further studies reveal that in individuals undergoing broad-based genetic testing with a kidney gene panel, variants in *SALL1* are rare, yielding a prevalence of 1:1,592 among patients tested for monogenic kidney disease. Usually, these patients (in 91%) progressed to CKD with decreased glomerular filtration rate and included diverse kidney features (agenesis/hypoplasia, focal segmental glomerulosclerosis, kidney cysts). Hearing loss/ear anomalies or anorectal malformations were present in about a third (not all having both), and only 18% had hand/thumb abnormalities. Only 14% of patients had the traditional “triad” called Townes-Brocks syndrome (11). As the variants are rare, this data is based on a relatively small set of patients. Nevertheless, they are consistent with findings in our patient.

On the contrary, auricular anomalies and appendages are more common, with an estimated incidence of external ear malformations in 1 per 6,000 to 6,830 newborns, associated with syndromes involving other organ systems in 30% (14). Most ear anomalies are therefore acquired and originate from exposures *in utero* (infections, medications, malnutrition, Rh incompatibility, hypoxia, bleeding, disturbances of metabolism) (14). Therefore, in the absence of cardinal features of a syndrome, the clinical suspicion for associated kidney disease is low.

Our case emphasizes the need for increased clinical suspicion of apparently isolated anomalies to think of other clinical manifestations, especially in external ear anomalies and kidney disease. Early diagnosis is important for comprehensive evaluation of potentially affected organ systems, genetic testing of family members and genetic counseling, early renal management, which can be crucial in slowing of CKD progression (15). Concerning CKD, ongoing monitoring and renal protective interventions are needed.

Otherwise, management of Townes-Brocks syndrome depends on clinical presentations and is usually based on multidisciplinary approach with involvement of several specialists, such as pediatric nephrologist for management of CKD, an ear-nose-throat (ENT) specialist for hearing aids and cosmetic surgical procedures, and orthopedic or general surgeons when limb or anorectal abnormalities are present. According to other manifestations, other specialists might be included as well, such as ophthalmologist for any eye-related manifestations, or neurologist when developmental delay is present etc.

## Conclusion

We confirmed Townes-Brocks syndrome in a 10-years-old girl. The suspicion of the genetic cause of the disease was established already with bilateral kidney hypoplasia; however, the history of auricular hypoplasia and appendages directed us to a specific genetic origin. We encourage kidney disease suspicion whenever ear anomalies are present despite absence of other possible malformations.

## Patient perspective

Children with apparently isolated ear anomalies should be screened for kidney disease as this is a possible association. Identification of a specific gene mutation allows comprehensive management of a patient with a reduction of unnecessary diagnostic procedures. Timely diagnosis of kidney disease allows early management of CKD in children, possibly prolonging their normal glomerular filtration rate and postponing the development of complications.

## Data Availability

The original contributions presented in the study are included in the article/Supplementary Material, further inquiries can be directed to the corresponding author.
